# Influence of Cross-Linking Agents on the Structure and Stability of Chitosan and Carboxymethyl Chitosan Thin Films

**DOI:** 10.3390/molecules31020272

**Published:** 2026-01-13

**Authors:** Katarzyna Lewandowska

**Affiliations:** Faculty of Chemistry, Nicolaus Copernicus University in Toruń, Gagarin 7, 87-100 Toruń, Poland; reol@umk.pl

**Keywords:** cross-linking, chitosan, carboxymethyl chitosan

## Abstract

Chitosan (CS) and carboxymethyl chitosan (CMCS) are polysaccharides valued for their biocompatibility, reactivity, and film-forming capabilities. This study compares the surface characteristics and stability of CS and CMCS thin films crosslinked with citric acid (CTA), polyethylene glycol diglycidyl ether (PEGDE), and glutaraldehyde (G). Flow behavior was assessed using steady-shear measurements, while film structure, morphology, and physical properties were analyzed by infrared spectroscopy, SEM, AFM, mechanical testing, and swelling experiments. Crosslinking generated new chemical bonds in both CS and CMCS films; however, interactions in CMCS did not result in stable cross-links and were comparatively weaker. These structural modifications influenced swelling behavior and enhanced stability, particularly in CS-based systems. Before neutralization, CS/PEGDE films exhibited the lowest swelling (67% ± 19) relative to unmodified CS (118% ± 25) and crosslinked samples such as CS/G2 (185% ± 30), CS/G1 (475% ± 88), and CS/CTA (520% ± 90). After neutralization, CS/G1 and CS/CTA maintained the highest swelling capacity. In contrast, CMCS films crosslinked with CTA and G1 dissolved rapidly in aqueous media due to high water uptake, while PEGDE- and G2-modified CMCS films demonstrated stability comparable to CS. Overall, the results highlight the superior stability and tunable surface properties of CS-based films, underscoring their potential for biomedical and packaging applications.

## 1. Introduction

Biopolymers such as cellulose, gums, chitin, or chitosan and their derivatives are well known for their high biocompatibility, biodegradability, inherent antibacterial activity and other biological properties [1,2,3,4,5,6,7]. Furthermore, these polysaccharides offer ecological safety, renewability, and sustainability. They are widely used to obtain biomaterials for environmental, biomedical, pharmaceutical, cosmetic, and other applications [8,9,10,11,12]. The main limitations of the wide use of these biopolymers are their deficient properties, such as weak mechanical and thermal stability, too rapid biodegradability, and high water sorption. Some modifications have been made to overcome these limitations and produce more thermally, mechanically and chemically stable biopolymer products. One of the common approaches to improve the fundamental properties and stability of materials based on polysaccharides is through chemical and/or physical modifications, including mainly crosslinking. Cross-linking is an effective method to improve the physicochemical properties of polysaccharide-based materials, making them valuable for biomedical, cosmetic and other applications [13,14,15]. The cross-linking process is carried out using both synthetic (e.g., glutaraldehyde, carbodiimide, boric acid) and natural (e.g., enzymes, genipin, tannic acid) cross-linking agents. Crosslinked chitosan and carboxymethyl chitosan materials hold significant potential for tissue engineering due to their enhanced properties and biocompatibility [16,17,18,19,20,21,22].

This study examines and compares the effects of different cross-linking agents on the morphology, structure, and surface properties of CS and CMCS thin films. The cross-linking agents investigated include citric acid (CTA), polyethylene glycol diglycidyl ether (PEGDE), and glutaraldehyde (G). Previous studies have shown that these agents effectively modify the properties of CS-based materials [16,23,24,25,26,27]. For instance, films crosslinked with citric acid exhibited enhanced water resistance, transparency, and improved thermal, mechanical, and antioxidant properties compared to neat chitosan films [16,23,25]. When PEGDE and G were used as cross-linkers [24,26], CS-based systems demonstrated a reduction in free amine groups, crystallinity, and hydrophilicity. Furthermore, desired levels of water sorption, mechanical strength, and other functional properties can be tuned by adjusting citric acid concentration and neutralization conditions. In addition to cross-linking, we employed a neutralization step with NaOH, as described in the literature [16,28,29,30], to further tailor CS film properties. For CMCS, glutaraldehyde alone or in combination with polymers (PVA, alginate) or calcium ions has been used as a cross-linker [31,32,33,34]. These studies demonstrated that cross-linked CMCS-based hydrogels and membranes are promising for applications in food technology, tissue engineering, wound dressings, and drug delivery. Previous studies [16,23,24,25,26,27] have investigated the effect of cross-linking agents, such as PEGDE, G, or CTA, on the properties of CS; however, the change in these properties after the neutralization process of cross-linked CS materials was not further assessed. Additionally, the same cross-linking agents were used to obtain cross-linked CMCS-based materials. Consequently, direct comparative studies examining the surface and structural behavior of CS and CMCS films modified with identical cross-linking agents are, to our knowledge, scarce or unreported.

The main objective of this research was to comprehensively evaluate the flow behavior, structure and surface characteristics of CS and CMCS films using steady-shear tests, scanning electron microscopy (SEM), atomic force microscopy (AFM), mechanical testing, swelling behavior analysis, thermogravimetric analysis (TGA), and infrared spectroscopy (IR). In CS systems, neutralization was applied alongside cross-linking. This process significantly reduces swelling and alters surface properties [28,29]. Notably, no previous reports have described the combined application of CTA, PEDGE, and G cross-linking with subsequent neutralization in CS films. By analyzing films before and after neutralization, we aimed to elucidate the individual and synergistic effects of these modifications. As neutralization is essential prior to rehydration of chitosan materials, our findings provide new insights into the stabilization and functionalization of CS and CMCS films. This knowledge may facilitate the design and development of innovative biomaterials for use in cosmetics, tissue engineering, and medicine.

## 2. Results and Discussion

### 2.1. Steady Shear Measurements

**Figure 1:**
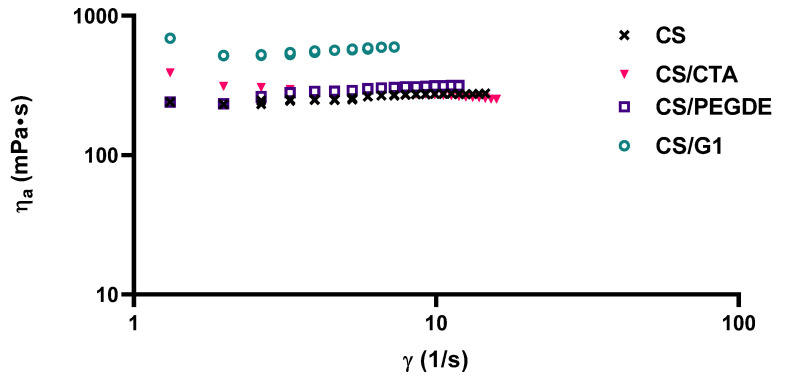
Viscosity curves of CS solutions with the addition of cross-linking agents at 25 °C.

Steady shear tests were carried out to evaluate the rheological behavior of the pure CS and CMCS solutions and their modified solutions by adding cross-linking agents. Figure 1 shows the viscosity curves as a function of shear rate for the CS solutions with and without cross-linking agents. All investigated solutions exhibited non-Newtonian behavior. For the pure CS solution and the one modified with PEDGE or G, the apparent shear viscosity slightly increased with an increasing shear rate, indicating a shear-thickening behavior. According to previous reports [35,36], this behavior is attributed to molecular conformational changes induced by flow and the association of polymer molecules. This phenomenon may be attributed to strong intermolecular interactions and complex formation between the active functional groups of CS within the network structure and those of PEGDE or G. These interactions facilitate the formation of a temporary network, thereby increasing the viscosity of the polymer solutions [25,36].

Furthermore, the apparent viscosity of CS/PEDGE and CS/G1 solutions was higher than that of the pure CS solution. In contrast, shear-thinning behavior was observed for the CS/CTA solution, where the apparent viscosity decreased as the shear rate increased. This behavior is associated with the alignment of polymeric chains, which are initially randomly distributed but become oriented under shear [25,35,36,37].

The power-law model was applied to evaluate the flow properties of modified CS solutions. The rheological parameters obtained from this model are summarized in Table 1. The R^2^ values were greater than 0.98, indicating that the viscosity curves were accurately described by the power-law model. As observed, in the pure CS solution and in the solutions modified with PEDGE or G, where shear-thickening behavior was evident, the flow behavior index (***n***) was greater than 1 [35,36,37]. In contrast, for the CS/CTA solution, the ***n*** value was below 1, indicating shear-thinning behavior. The increase in the ***n*** value for the CS/PEGDE and CS/G solutions suggests the formation of a network structure due to the cross-linking of chitosan chains, which enhances the viscosity of the polymer solutions. Under flow conditions, networks or entanglements of unfolded macromolecules may form, influencing the flow behavior of the modified CS solutions [36]. Conversely, in the CS/CTA solution, shear forces disrupt hydrogen bonds and electrostatic interactions between the CS and CTA chains, leading to a shear-thinning effect and an ***n*** value below 1.

**Figure 2:**
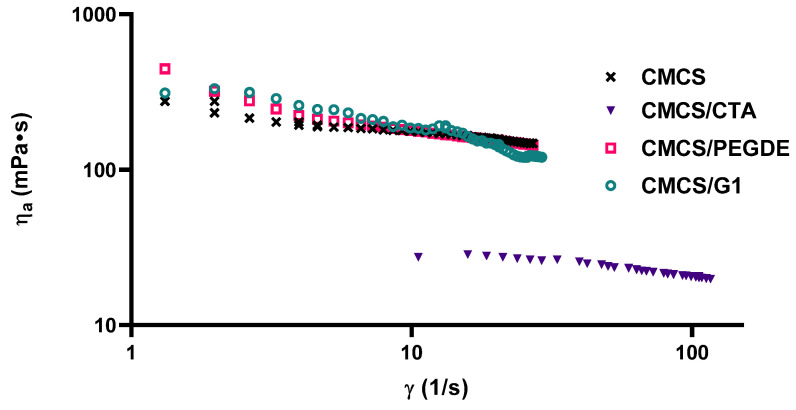
Viscosity curves of CMCS solutions with the addition of cross-linking agents at 25 °C.

Figure 2 presents the rheological behavior of CMCS and its modified solutions. The apparent shear viscosity of both pure and modified CMCS solutions decreased with increasing shear rate, demonstrating shear-thinning behavior. At low shear rates (<10 s^−1^), the higher apparent viscosity of CMCS/PEGDE and CMCS/G1 solutions can be attributed to the formation of intermolecular aggregates and stronger entanglements among CMCS molecules induced by the addition of PEGDE and G1. For example, at 1.32 s^−1^, the η_a_ value increased from 276 mPa·s for the pure CMCS solution to 312 and 444 mPa·s for the CMCS/G1 and CMCS/PEGDE solutions, respectively. As reported previously [36,37], this effect can be explained by reinforced internal resistance to flow, resulting from stronger chain entanglements and preliminary gelation of CMCS solutions. Gelation was particularly pronounced in CS and CMCS solutions containing G2, where flow measurement could not be performed. In contrast, the addition of CTA to the CMCS solution significantly reduced the apparent viscosity (Figure 2). This effect may be due to the absence of molecular aggregation and weaker repulsive forces between CMCS and CTA molecules. According to the power-law model (Table 1), all pure and modified CMCS solutions exhibited shear-thinning behavior, with flow behavior index (***n***) values ranging from 0.62 to 0.86. Among the tested samples, CMCS/PEGDE and CMCS/G1 showed the highest consistency index (***k***) and the lowest ***n*** values in the studied solutions. Conversely, the consistency index of the CMCS solution decreased substantially upon addition of CTA (Table 1), suggesting that CTA reduces flow resistance.

### 2.2. Mechanical Tests

**Figure 3:**
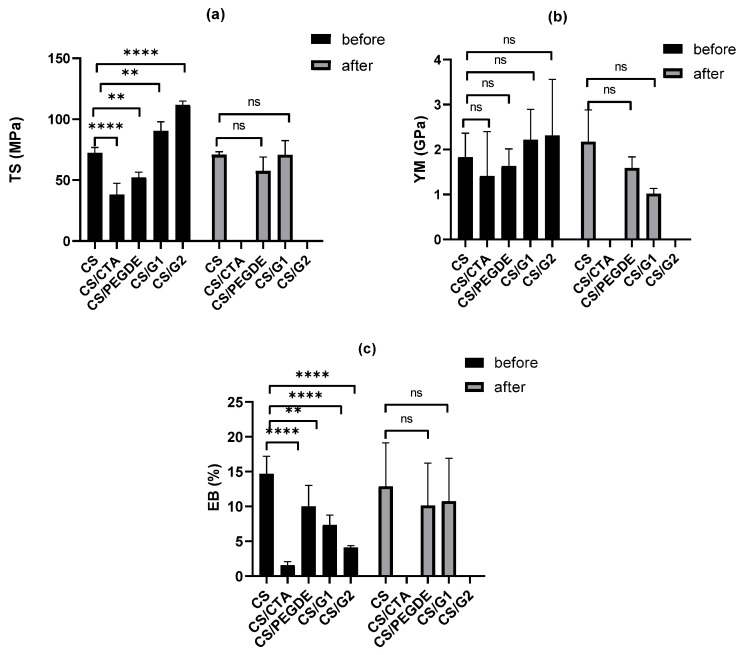
Mechanical properties of films based on CS (**a**) TS, (**b**) YM, and (**c**) EB, before and after alkaline treatment, *n* = 5, mean ± SD (standard deviation), error bars represent SD, ns—not significant, **—0.001, ****—0.0001.

The impact of cross-linking agents on the mechanical properties of CS and CMCS films—namely tensile strength (TS), Young’s modulus (YM), and elongation at break (EB)—was investigated. Figure 3 presents the mechanical parameters of pure CS film and its modified films before and after neutralization.

Before alkaline treatment, the modified CS films exhibited YM values ranging from 1.4 GPa to 2.3 GPa (Figure 3), compared to 1.7 GPa for the pure CS films. The lowest YM values were observed for CS/CTA and CS/PEGDE films, while the highest values were obtained for CS/G films. The tensile strength (TS) of the modified films varied between 32.3 MPa and 111.7 MPa, with the highest TS also found for films containing G. For the pure CS film, the elongation at break (EB) was 14.7%. Upon addition of cross-linking agents, all films showed reduced EB values (1.6−10.0%), with the lowest recorded for CS/CTA. As reported in previous studies [38,39], the observed reduction in deformation directly results from the increased cross-link density induced by the cross-linking agents. After alkaline treatment, changes in the mechanical properties were observed for CS and CS/G1 films, whereas CS/PEGDE films maintained nearly the same values within experimental error. The stability of CS/PEGDE properties suggests effective cross-linking. For the pure CS film, TS remained constant, YM increased by ~30%, and EB slightly decreased. In contrast, alkaline treatment improved the elasticity of the CS/G1 film, as reflected by decreased TS and YM values and an increased EB. CS/G2 and CS/CTA films were too brittle to measure, as they cracked during cutting. The observed changes in mechanical properties are associated with the removal of residual acetic acid as well as alterations in the surface topography and physical characteristics of the modified films [29,36]. Previous studies have reported that neutralization promotes polymer chain rearrangement and tighter packing. Moreover, this effect may partially arise from water absorption during the neutralization step, with retained water becoming trapped between polysaccharide chains and acting as a plasticizer.

**Figure 4:**
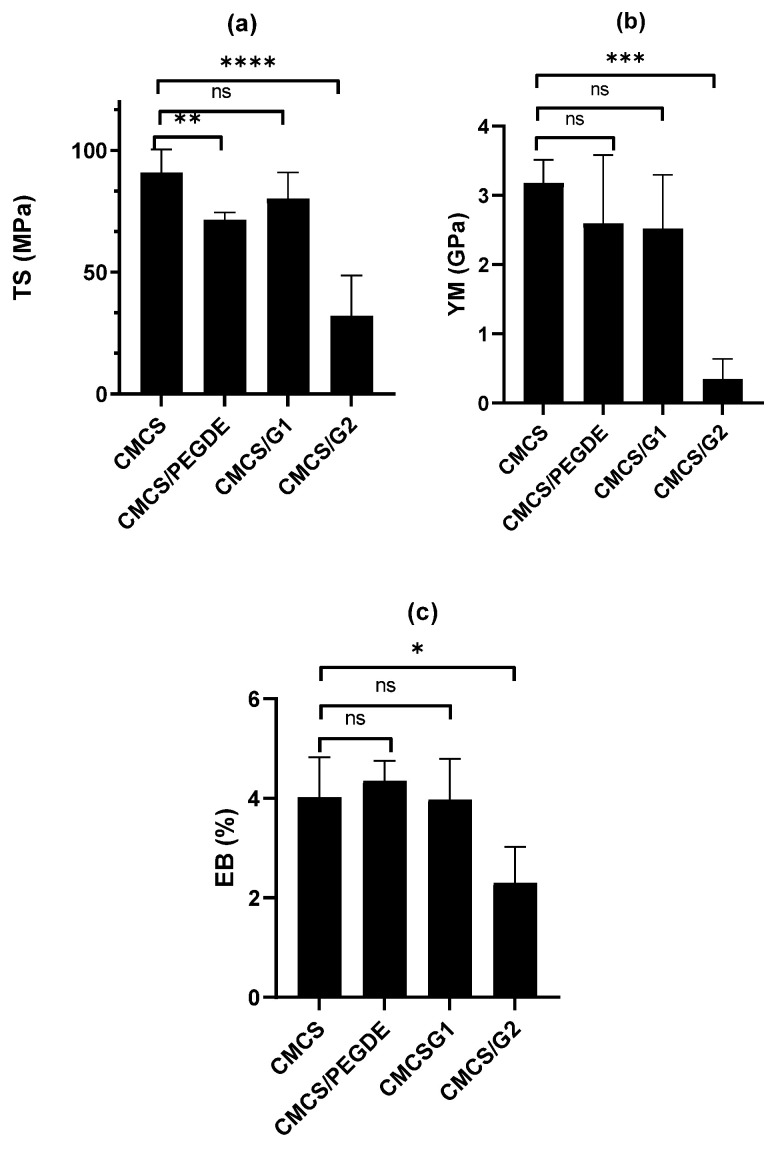
Mechanical properties of films based on CMCS: (**a**) TS, (**b**) YM, and (**c**) EB, *n* = 5, mean ± SD (standard deviation), error bars represent SD, ns—not significant, *—0.01, **—0.001, ***—0.0005, ****—0.0001.

Figure 4 shows the mechanical properties of CMCS films and those modified with cross-linking agents. The tensile strength (TS) of pure CMCS films (91 MPa) was higher than that of the modified films (72 MPa for CMCS/PEGDE and 80 MPa for CMCS/G1). For both CMCS/G1 and CMCS/PEDGE films, the Young’s modulus (YM, ~2.5 Gpa) and elongation at break (EB, ~4%) remained nearly unchanged compared to pure CMCS films, within experimental error. However, a further increase in cross-linker content, as observed in CMCS/G2, resulted in a significant deterioration of mechanical properties (Figure 4). Owing to over-crosslinking, the tensile strength and Young’s modulus of CMCS/G2 films (32 MPa and 0.4 Gpa, respectively) were markedly lower than those of CMCS/PEGDE and CMCS/G1 films. The high G content in CMCS/G2 reduced polymer chain flexibility, leading to decreased strength and elongation and rendering the films more brittle [16].

### 2.3. Morphological Studies

**Figure 5:**
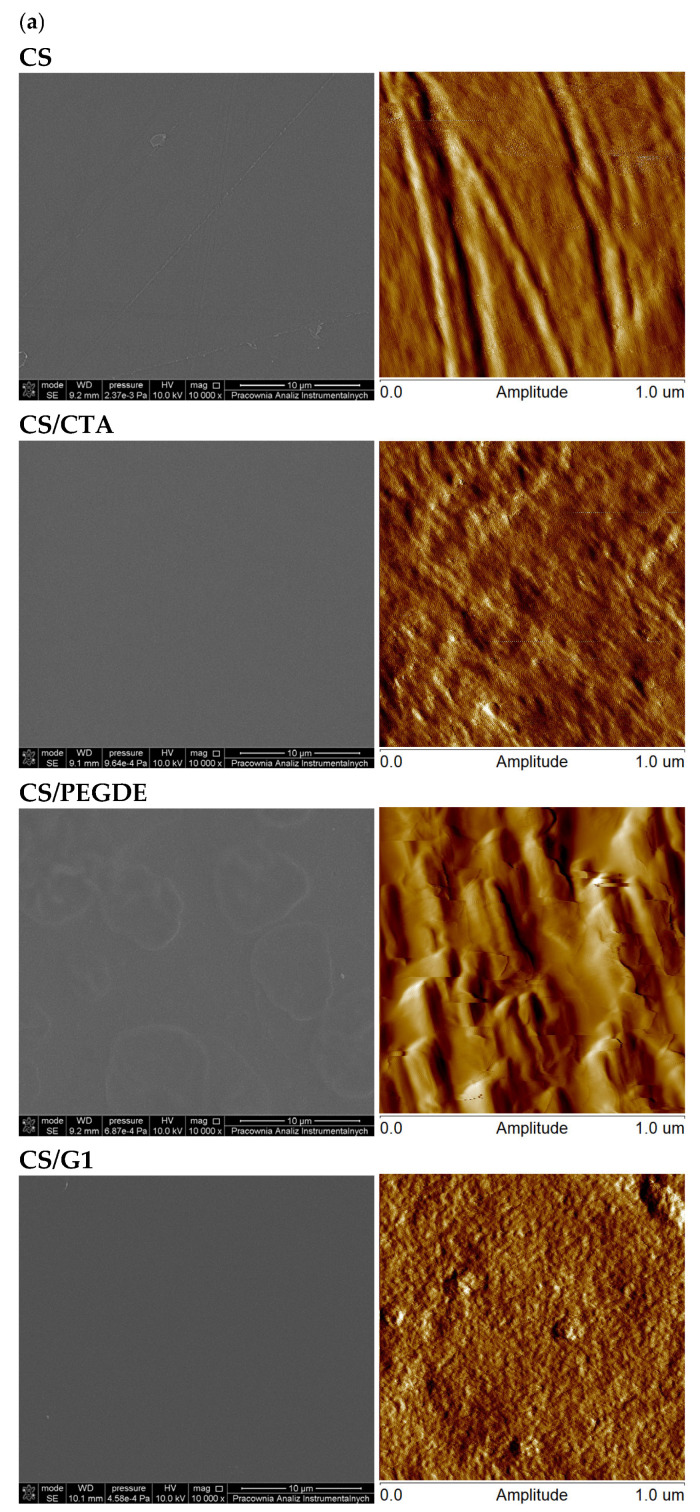

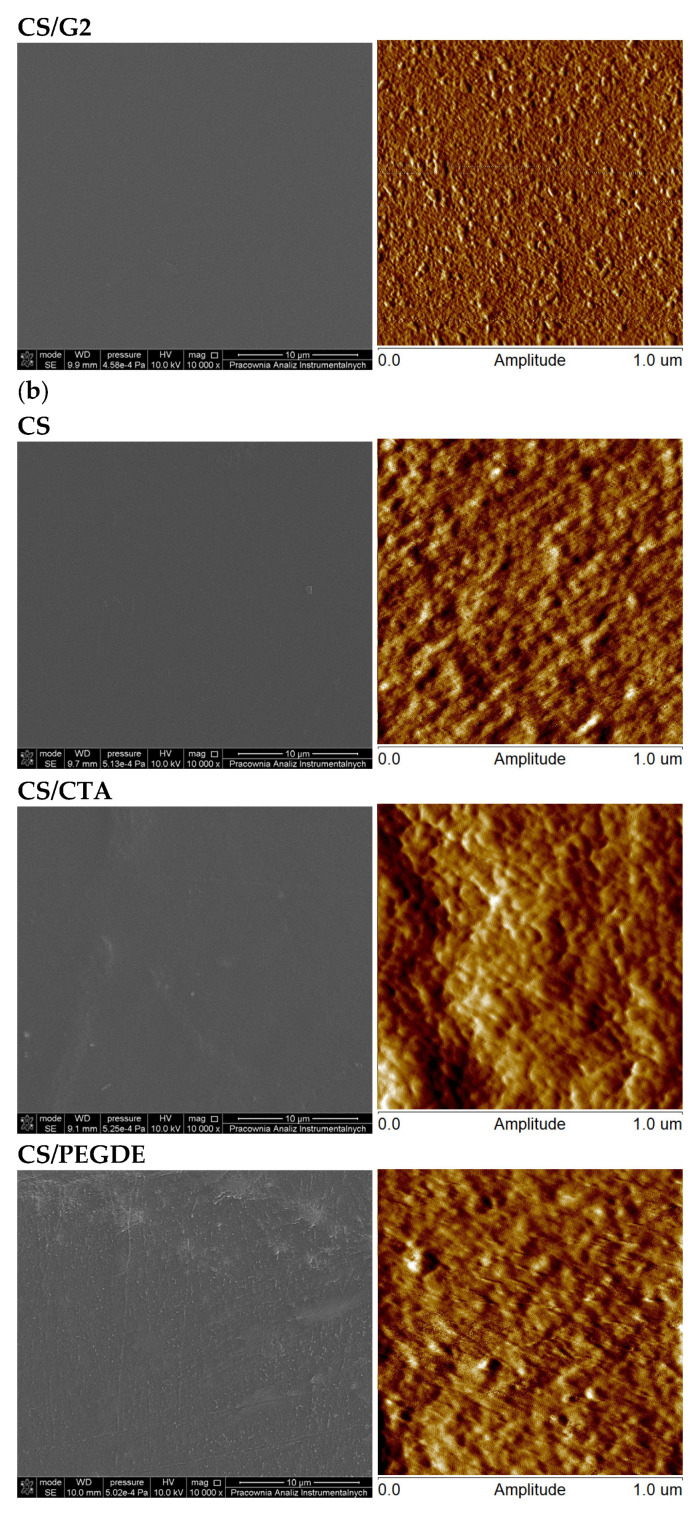

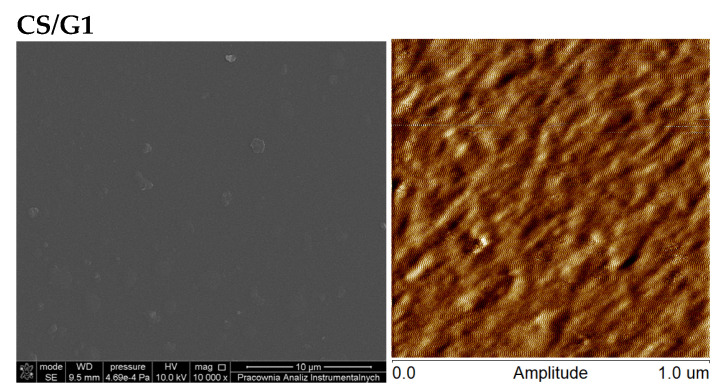
Representative SEM and AFM images of thin films based on CS (**a**) before and (**b**) after NaOH treatment.

Figure 5 and Table 2 present representative SEM and AFM images of the surface morphologies, along with roughness parameters of CS films before and after neutralization. Pure CS, CS/CTA and CS/G films exhibited relatively compact and smooth surfaces, with roughness values of ~3.6 nm for CS, 1.7 nm for CS/CTA, and 1.5 nm for CS/G1 (Table 2). In contrast, CS/PEGDE films showed pronounced changes in surface morphology, becoming rougher, with uniformly distributed granular micropatterns. This new phase formation is attributed to surface modification induced by the addition of PEGDE. Among all studied films, PEGDE produced the most significant effect on surface morphology, as reflected by the highest roughness value.

After NaOH treatment (Figure 5b), SEM analysis revealed no major changes in the surface morphology of CS and CS/G1 films. However, the roughness of the CS/G1 film was approximately half that of the pure CS film, indicating improved homogeneity of CS crosslinked with G1 compared to other films. This observation is consistent with previous reports [24,39], which described reduced roughness and increased surface uniformity following chemical modification. For CS/CTA and CS/PEGDE films (Figure 5b), neutralization resulted in the appearance of holes on the surface. These defects are likely caused by solvent leaching and the release of unbound or weakly hydrogen-bonded CTA and PEGDE. For CS/CTA films, the surface roughness increased following neutralization, as evidenced by the corresponding rise in roughness parameters presented in Table 2.

**Figure 6:**
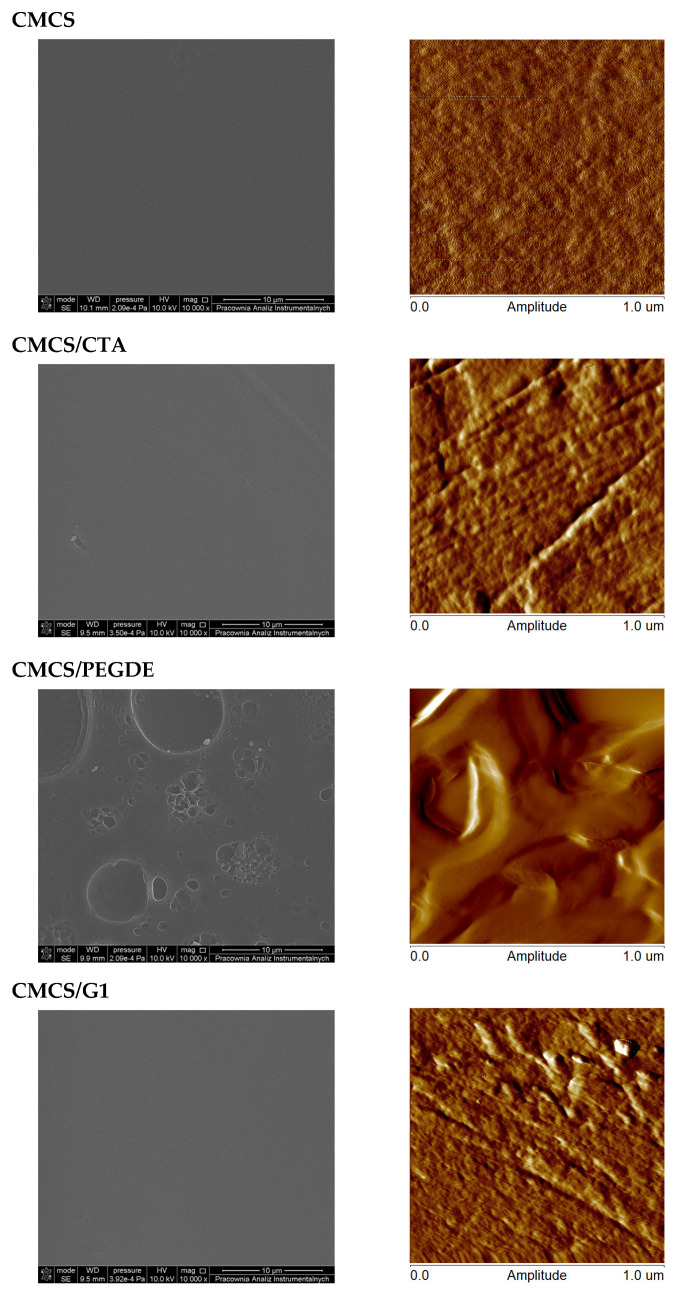

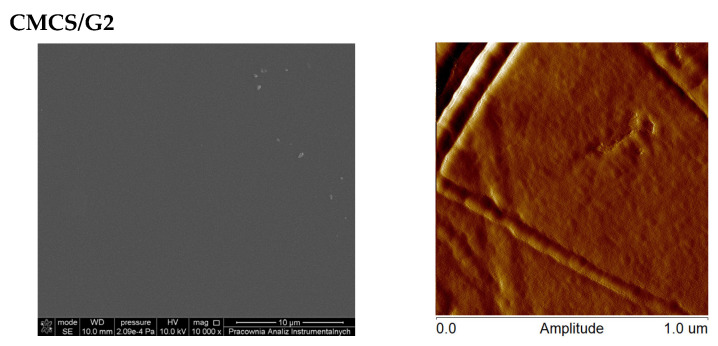
Representative SEM and AFM images of thin films based on CMCS.

Pure CMCS and CMCS/PEGDE films (Figure 6) exhibited a granular texture, while CMCS/CTA and CMCS/G films displayed surfaces characterized by channel-like structures. These microstructural modifications influence both the mechanical properties and the swelling behavior of films. Moreover, the roughness values of films containing cross-linking agents (Table 2) were higher than those of the pure CMCS film. This increase can be attributed to interactions between the polymer matrix and cross-linking agents. Such interactions affect the morphological and mechanical properties of CMCS films, leading to distinct surface morphologies depending on the type of cross-linker added.

### 2.4. Swelling Behavior

**Figure 7:**
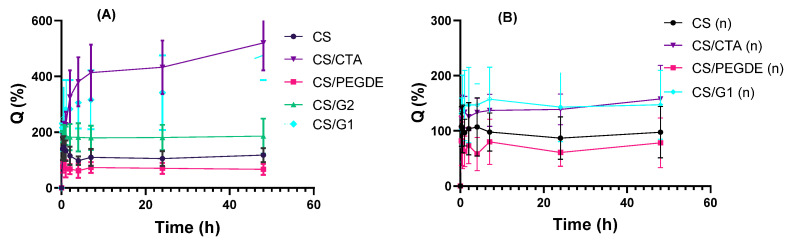
Swelling ratio of thin films based on CS (**A**) before and (**B**) after NaOH treatment in the PBS solution at 37 °C.

Swelling behavior is a key characteristic of polymer materials and plays a crucial role in determining their suitability for biomedical applications. It is well established that the swelling behavior of a material depends on its chemical composition, the nature of the polymer–solvent interactions, the flexibility of the polymer chains, the molar mass of the polymer, and the presence of chemical cross-linkers [38,40]. The swelling ratio of CS and its modified films, both before and after NaOH treatment, was evaluated by measuring their weights after immersion in PBS solution at 37 °C. The swelling curves are presented in Figure 7.

Before NaOH treatment, almost all swelling curves show that the swelling ratio reaches a maximum after half an hour and then gradually decreases with time, except for the CS/CTA and CS/G1 films. After immersion in PBS, the swelling ratio of the CS films decreased from ~145% after half an hour to ~118% after 48 h. A progressive increase in swelling ratio values was observed for the CS/CTA and CS/G1 films, with the highest values recorded after 48 h at 520% and 478%, respectively. These observed changes in swelling ratios are attributed to intense initial swelling of the films, followed by gradual degradation over time. These phenomena contribute to the variability observed in the data, as reflected by the error bars shown in Figure 7. The CS/PEGDE film swelled by approximately 82% after half an hour, decreasing to around 67% at 48 h. For the CS/G2 films, the swelling ratio remained practically constant at approximately 185%. In addition, the results presented in Figure 7 reveal a pronounced difference in swelling ratio between CS/G1 and CS/G2, which is attributable to the concentration of the cross-linking agent in the polymer matrix and the resulting cross-link density. CS/G1 films exhibit higher swelling at lower G concentrations due to a lower degree of cross-linking, reduced cross-link density, and greater network flexibility. In contrast, CS/G2 films, containing a high G concentration, display a higher degree of cross-linking and cross-link density, resulting in a stiffer network and a markedly lower swelling ratio. As reported previously [41,42], increasing the cross-linker content enhances the cross-linking degree of the polymer matrix, thereby hindering water permeation into the film. After NaOH treatment, it is evident that CS and its modified films reached equilibrium after half an hour, followed by a slight decrease to a steady value. The differences in swelling ratio between the films were relatively small. Among them, the highest swelling was observed for the CS/G1 film (147%); however, this value was lower than that observed before NaOH treatment.

**Figure 8:**
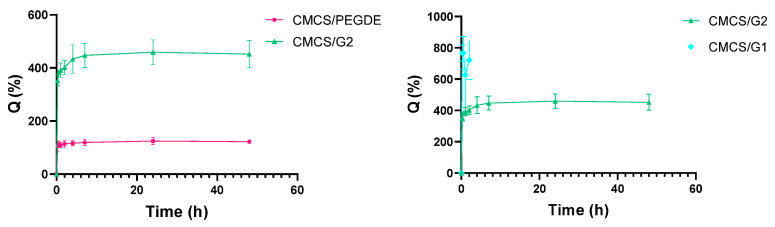
Swelling ratio of thin films based on CMCS in the PBS solution at 37 °C.

Figure 8 presents the swelling ratio values for CMCS/PEGDE and CMCS/G films. As can be seen, the swelling curves for CMCS/PEGDE and CMCS/G2 films show that the swelling ratio reaches a maximum after two hours and then remains practically constant. The values obtained for CMCS/PEGDE are significantly lower (120%) than for CMCS/G2 films (450%), similar to the results for the crosslinked CS films. For the CS/CTA and CS/G1 films, hydrogel formation was observed after 15 min and two hours (Figure 8), respectively. Thus, for these films, the modifications and crosslinking were minimal. Upon introduction into the PBS solution, the samples absorbed water rapidly and dissolved, which made it impossible to determine their swelling ratio.

### 2.5. Thermogravimetric Analysis

**Figure 9:**
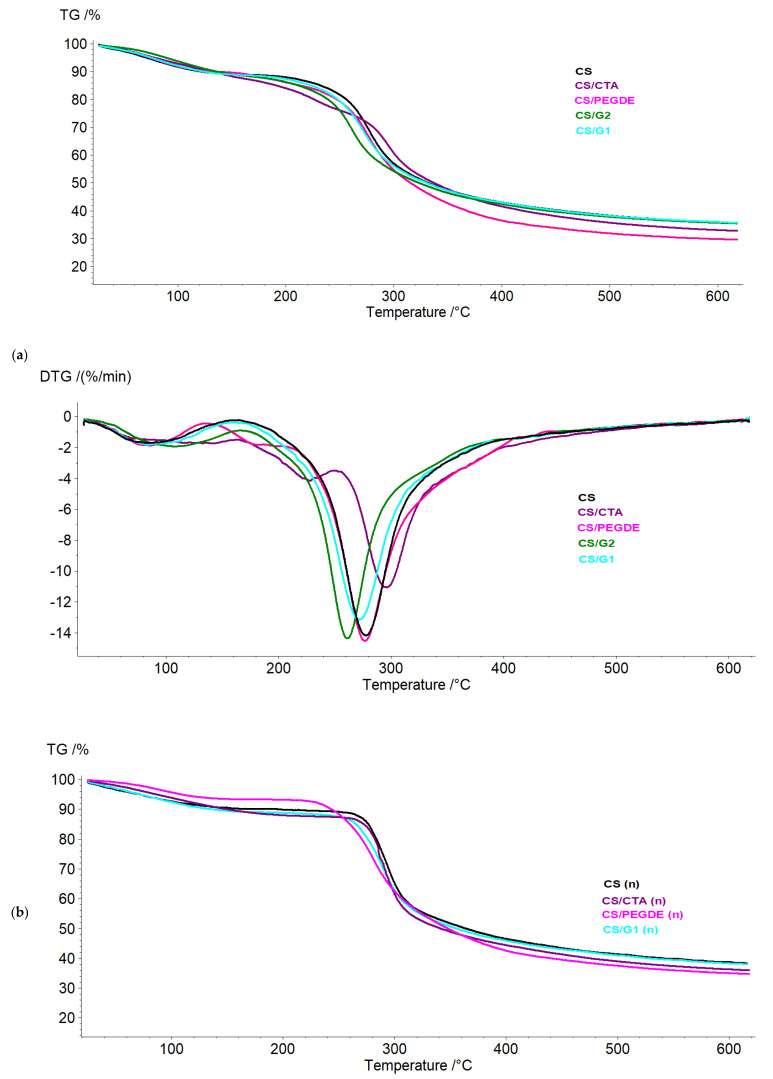

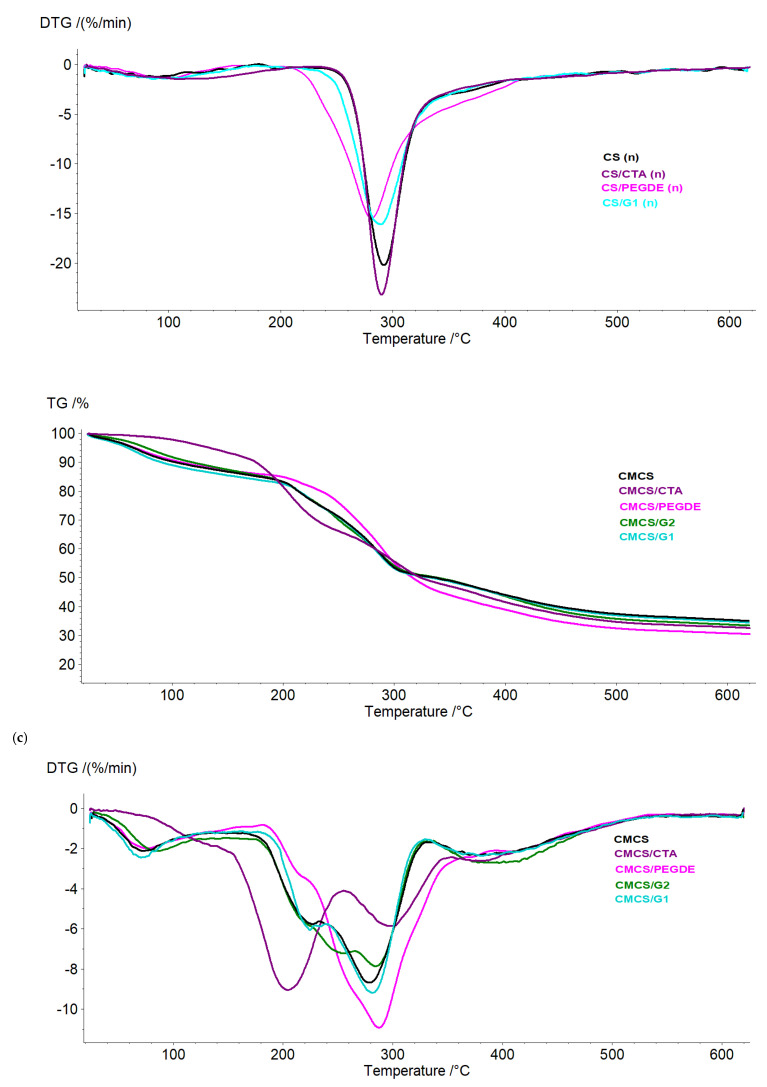
TGA thermograms of thin films based on CS (**a**) before and (**b**) after NaOH treatment, and (**c**) CMCS.

The thermal characteristics of modified films should be considered to determine the feasibility of thermal treatment, sterilization, stability, and further modification. Figure 9 presents the TGA results for pure CS and CMCS films, as well as their modified films, before and after NaOH treatment. For pure CS, a prominent decomposition peak was observed in the 220–380 °C range, with a peak temperature of about 277 °C (Figure 9a). After neutralization, the first stage, up to 220 °C, corresponded to the removal of residual acid, and the maximum weight loss shifted toward to a higher value, centered at 292 °C (Figure 9b). These results for CS are consistent with previous reports [24,30]. For the CS/PEGDE and CS/CTA films, a decrease in weight loss associated with adsorbed water in the first stage was observed compared to the pure CS film. Additionally, the primary decomposition stage shifted to higher temperatures for the CS/PEGDE and CS/G films, which may be attributed to cross-linking between CS and PEGDE or CTA, reducing water absorption in the films. In the case of the CS/G film, only a minor change in the thermal curve was detected.

Analysis of the thermograms of all films after neutralization (Figure 9b), showed that the stages up to 240 °C corresponded to the removal of residual acetic acid and unbound cross-linking agents. In the subsequent phase of maximum weight loss, no significant shift toward higher temperatures was observed for the modified films compared to the pure CS film. This finding suggests that the cross-linking interaction did not enhance the thermal stability of the modified films. The reason for the slight changes in thermal stability is the nature of the cross-links formed, which decompose at high temperatures. However, in the cases of the CS/PEGDE and CS/G1 films, the main decomposition peak appeared broader, which may be attributed to stronger cross-linking within these materials.

Pure CMCS exhibited thermal stability up to 200 °C, with a 12% weight loss, which could be attributed to the evaporation of both loosely adsorbed and bound water. The main decomposition peak appeared in the 200–300 °C range, centered at 285 °C. For the CMCS films modified with PEGDE or G (Figure 9c), the addition of these substances did not significantly influence the thermal stability of the CMCS film, as evidenced by only slight variations in the peak temperatures of the corresponding thermal stages. Similarly to CS, the CMCS/G and CMCS/PEGDE films exhibited an apparent broadening of the central decomposition peak. In contrast, substantial changes in thermal decomposition were observed for the CMCS/CTA film, which displayed three distinct weight-loss stages. In the first stage, a reduction in mass loss due to adsorbed water was observed compared to the pure CMCS films. Moreover, the main decomposition stage in this film occurred at a much lower temperature (207 °C), corresponding to a 28% mass loss. The third decomposition stage was recorded at 298 °C, with a mass loss of 19%. These shifts can be attributed to intermolecular interactions between CMCS and CTA, which altered the thermal decomposition behavior. As previously reported [43], the crystal structure of CTA, hydrogen bonding, and ionic interactions between CTA and CMCS likely contribute to these observed changes.

### 2.6. Infrared Spectroscopy

**Figure 10:**
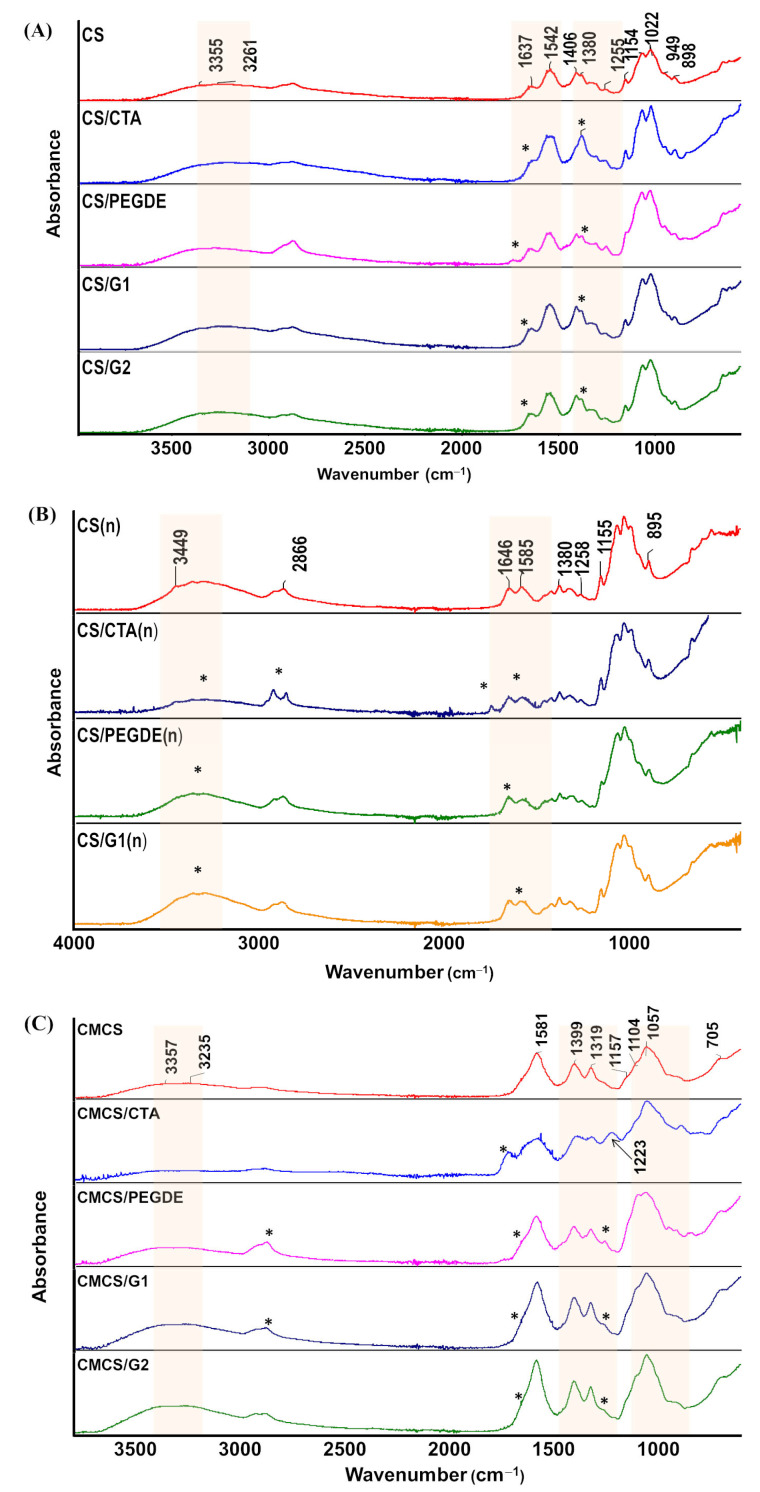
Infrared spectra of (**A**) thin films based on CS before and (**B**) after NaOH treatment, and (**C**) CMCS, * and red area—indicate changes in the characteristic bands.

Infrared spectroscopy in ATR mode was used to study the interaction between CS or CMCS and cross-linking agents. The corresponding infrared spectra are presented in Figure 10. The spectra of pure CS and CMCS exhibit a series of characteristic bands in the 3000–3400 cm^−1^ region, corresponding to O–H and N–H stretching modes, and in the1000–1200 cm^−1^ range, associated with skeletal vibration of saccharide rings [23,24,35].

In contrast, within the 1700–1300 cm^−1^ region, significant differences in the positions of characteristic bands are observed between CS and CMCS, reflecting the presence of different functional groups. For the CS film (Figure 10A), the absorption band at 1637 cm^−1^ is attributed to C=O vibrations of the amide I band. The amide II and III bands appear at 1542 cm^−1^ and 1380 cm^−1^, corresponding to C–N stretching and N–H bending vibrations, respectively [23,24,35]. After alkaline treatment (Figure 10B), changes are observed in the shape and intensity of the amide I, II, and III bands, as well as in the 1200–1500 cm^−1^ region, due to modifications of the amine groups [28,29,30]. The neutralization process reduces the hydration shell surrounding the amine groups, thereby facilitating the formation of new interactions within the polymer chains [28,29].

In the case of the CMCS film, the spectrum exhibited bands at 1581 cm^−1^ and 1399 cm^−1^, corresponding to the asymmetric and symmetric stretching vibrations of the carboxylate C=O groups, respectively [35,38]. The absence of characteristic bands in the 1720–1750 cm^−1^ region indicates that carboxylic acid groups are not present, suggesting that CMCS exists in its sodium salt form. The peaks at 1319 cm^−1^ and 1057 cm^−1^ are attributed to C–N stretching vibration and alkoxy (–C–O) vibrations, respectively [38].

For the CS/CTA film, the infrared spectra recorded before and after NaOH treatment reveal changes in the intensity and shape of the amide bands in the 1650–1300 cm^−1^ region, as well as in the 3000–3500 cm^−1^ range. These variations suggest weak hydrogen-bonding interactions between the amino and hydroxyl groups of CS and the hydroxyl or carboxyl groups of CTA. After neutralization, the spectral changes become less pronounced, likely due to the partial removal of CTA during the process. When CS is cross-linked with G, the intense band at 1650 cm^−1^ represents a combination of amide I and imine bonds. Notably, the amide II band appears more intense than the amide I band before alkaline treatment (Figure 10A, marked with an asterisk). Following alkaline treatment, the intensities of these bands become comparable. In the case of the CS/PEGDE film, a new peak appears at 1734 cm^−1^, which disappears after neutralization. As reported previously [40], this band—observed only in the reaction mixture of CS with PEGDE in acetic acid—is attributed to ester formation between acetic acid and PEGDE. This side reaction occurs without the involvement of CS, as the 1734 cm^−1^ band is eliminated after treatment with ethanol or NaOH, consistent with both this study and the literature [44]. Additional changes in the intensity and shape of the amide bands (1650–1300 cm^−1^) and the hydroxyl stretching region (3500–3000 cm^−1^) in Figure 10B (marked with an asterisk) further support the participation of amine and hydroxyl groups in cross-linking reactions with the epoxide functional groups of PEGDE [23,24,43,45,46].

For CMCS films modified with cross-linking agents, the characteristic bands in the 1700–1500 cm^−1^ and 1200–1000 cm^−1^ regions (Figure 10C, marked with an asterisk) shift to lower wavenumbers and become broader. These changes indicate the formation of hydrogen bonds and ionic interactions between CMCS and the cross-linking agents. Additionally, a new peak at 1223 cm^−1^ appears, particularly in the CMCS/CTA film. As reported by Wen et al. [38], this peak is attributed to the protonation of –NH_2_ groups in the CMCS chains, leading to the formation of –NH_3_^+^ and its electrostatic interaction with –COO^−^ groups. However, these interactions do not result in stable cross-links, as the CMCS/CTA films absorb water and dissolve when immersed in PBS solution.

## 3. Materials and Methods

### 3.1. Materials

CS powder, with a viscosity average molar mass of 984 kg/mol and a degree of deacetylation of 77% (estimated through viscometric technique and potentiometric titration, respectively), was purchased from the Marine Fisheries Research Institute (Gdynia, Poland). CMCS was synthesized using a previously reported method, in which CS powder was treated with monochloroacetic acid in an alkaline medium at 55 °C [35,47,48]. Briefly, 2 g of CS was mixed with 2.7 g of sodium hydroxide in 4 mL of water and 16 mL of isopropanol, and the resulting solution was left at room temperature for 1 h. Subsequently, 3 g of monochloroacetic acid in 4 mL of isopropanol was added dropwise to the mixture over 30 min. The mixture was refluxed in a reflux condenser at 55 °C for 4 h. Ethyl alcohol (80%) was added, and the solid product was filtered and rinsed with 80% ethyl alcohol to desalt and dewater. The obtained product was in the sodium salt form and vacuum-dried at 25 °C. CMCS was characterized using viscometric techniques, potentiometric titration analysis, and infrared spectroscopy. CMCS was characterized using viscometric techniques, potentiometric titration, and infrared spectroscopy. It exhibited a carboxyl group content of 64% and a viscosity-average molar mass of 310 kg/mol. Viscosity-average molar masses were calculated using the Mark–Houwink–Sakurada equation [49]. Intrinsic viscosities ([η]) were measured by dilute solution viscometry using an Ubbelohde capillary viscometer, yielding [η] = 677.9 cm^3^/g for CS and [η] = 245.5 cm^3^/g for CMCS. The Mark–Houwink parameters for CS were obtained from the literature [50] and were K = 0.00181 cm^3^/g and a = 0.93 at 25 °C in 0.1 mol/dm^3^ CH_3_COOH/0.2 mol/dm^3^ NaCl. For CMCS in 0.1 mol/dm^3^ NaCl, the parameters were K = 7.92 × 10^−4^ cm^3^/g and a = 1.00 at 30 °C [51].

PEGDE (M_n_ 500) and G solution (50% in H_2_O) were purchased from Sigma-Aldrich (Poznan, Poland). Acetic acid and CTA were obtained from Chempur (Piekary Śląskie, Poland). All chemicals were of analytical grade and used as received, without further treatment, and were obtained from POCh (Avantor, Gliwice, Poland) and Chempur (Piekary Śląskie, Poland).

### 3.2. Film Preparation and Neutralization

CS was dissolved in 0.1 mol/L acetic acid at a concentration of 2% *w*/*v*. CMCS was dissolved in distilled water at 5% m/v at room temperature using a magnetic stirrer. Both CS and CMCS solutions were stirred continuously for 24 h until the polymer was fully dissolved. In this study, the cross-linking agents were selected based on references [23,24,25,26] for chitosan materials and were similarly applied to CMCS for comparative purposes. Mixture solutions containing cross-linking agents were prepared by blending aqueous CS or CMCS solutions (35 mL) in different weight proportions, as detailed in Table 3.

The films were cast into square plastic Petri dishes (10 × 10 × 1.5 cm) and allowed to dry at room temperature for 72 h. After drying, the polymer films were peeled off and prepared for further analysis. For CS materials, the films were immersed in a 1% NaOH solution for 15 min to neutralize the polymer matrix and remove excess acid. The films were then washed several times with distilled water until neutrality was achieved and left overnight. Finally, they were dried again.

### 3.3. Steady Shear Measurements

Steady shear tests were carried out on a ROTAVISC lo–vi complete rotational viscometer equipped with concentric cylinders (IKA-Werke Gmbh and Co. KG, Strufen, Germany) at different temperatures (25–35 °C). The apparent shear viscosity (η_a_) was determined as a function of shear rate (γ) from 1.3 to 100 1/s. The rheological data were fitted and analyzed according to the power-law model using the method described in previous reports [35,36].

### 3.4. Mechanical Tests

To investigate the mechanical properties of films, the tensile strength (TS, MPa), Young’s modulus (YM, GPa), and elongation at break (EB, %) were determined as a function of displacement by used force using Zwick Roell (Ulm, Germany) under a static load of 10 kg and a crosshead speed of 50 mm/min at room temperature. The testing was conducted following the standard method [52]. Samples were cut into the shape of paddles (width 4 mm at the center and a thickness ranging from 0.04 to 0.05 mm). A total of 5 samples of each type of film were tested. Statistical analysis was performed using GraphPad Prism 8.2.1 (GraphPad Software, San Diego, CA, USA). Results are presented as mean ± standard deviation (SD). One-way analysis of variance (ANOVA) was used to assess statistical differences, and data were also reported as mean ± standard error. Differences were considered statistically significant at *p* < 0.05.

### 3.5. Scanning Electron Microscopy (SEM)

A scanning electron microscope (Quanta 3D FEG, D9399, FEI, Eindhoven, The Netherlands) was used to observe the surface morphology of the films. To ensure conductivity for electron beam interaction, the films were coated with a thin layer of silver. The samples were observed at an accelerating voltage of 10.0 kV, a working distance of 9.2 mm, and a magnification of 10,000×.

### 3.6. Atomic Force Microscopy (AFM)

An atomic force microscope (Nanoscope IIIa Multimode Scanning Probe Microscope, Digital Instruments, Veeco Metrology Group, Santa Barbara, CA, USA) was used to analyze the images, operating in tapping mode at room temperature in an air atmosphere. The roughness parameters were calculated from AFM images (5 μm × 5 μm) as the root mean square (R_q_) using NanoScope Analysis v1.40 software (Bruker, Ettlingen, Germany). A standard AFM probe with pyramidal silicon tips (8 nm radius), a spring constant of 16 N/m, and a resonance frequency of 300 kHz was used (MikroMasch Europe, SPM Probes and Test Structures, Wetzlar, Germany).

### 3.7. Swelling Behavior

The swelling behavior of the films was studied in phosphate-buffered saline (PBS) at 37 °C using a gravimetric technique. Samples measuring 1 cm × 1 cm were first dried in a vacuum oven at 45 °C for 48 h. They were then immersed in PBS (pH 7.4) at 37 °C [36,44]. After incubation at specified intervals (0.25, 0.5, 1, 4, 8, 24, and 48 h), the films were gently blotted between two sheets of paper and weighed. The swelling ratio was calculated using Equation (1):

(1) $$\mathrm{Swelling}\;\mathrm{ratio}\;(\%)\;=\;\frac{\left(w_{1}-w_{0}\right)}{w_{0}}$$

where w_1_ is the weight of the wet film, and w_0_ is the weight of the dry films. Five samples of each film type were tested, and the mean values were taken as the swelling ratio.

### 3.8. Thermogravimetric Analysis (TGA)

A Jupiter STA 449 F5 thermal analyzer (Netzsch, Selb, Germany) was used to determine the decomposition temperature of the films. The samples were heated from 25 °C to 600 °C at a rate of 20 °C/min under a nitrogen atmosphere. The thermogravimetric (TG) and derivative thermogravimetric (DTG) thermograms of films were recorded.

### 3.9. Infrared Spectroscopy

A Nicolet iS10 spectrometer (Thermo Scientific, Waltham, MA, USA) equipped with attenuated total reflectance (ATR) mode and a diamond crystal was used to collect infrared spectra of the films. Spectra were recorded in the range of 4000–600 cm^−1^ with a resolution of 2 cm^−1^, and 100 scans were performed for each sample.

## 4. Conclusions

This study examined the effects of various cross-linking agents on the morphology, structure, and surface properties of CS and CMCS thin films. For CS-based films, both structural and surface properties were analyzed before and after the neutralization process. IR spectroscopy confirmed the formation of new bonds resulting from interactions between CS and the cross-linkers, as well as between CMCS and the cross-linkers. However, in the case of CMCS/CTA and CMCS/G1, these interactions did not lead to stable cross-links, as the modified films absorbed water and dissolved upon immersion in PBS solution. The structure and morphology of CS-based thin films were significantly influenced by cross-linking and neutralization, which in turn affected their swelling behavior. Neutralization induced surface changes and reduced roughness in CS, CS/PEGDE, and CS/G1 films, whereas roughness increased in CS/CTA films. Furthermore, a reduction in swelling capacity was observed, which is advantageous for biomedical and food packaging applications. Overall, CS-based materials subjected to cross-linking and neutralization exhibit strong potential for applications in biomedical, cosmetic, and packaging fields. For glutaraldehyde, it is crucial to balance cross-linker concentration with the desired material properties. Our results on PEGDE- and G2-cross-linked CMCS thin films indicate that these systems could serve as a foundation for further studies and the development of materials with biomedical and cosmetic potential, pending toxicity assessment. In contrast, the structural and surface modifications observed in CMCS/CTA films, along with their propensity for hydrogen bonding and ionic interactions, suggest that further optimization is required. Future research should therefore focus on enhancing the cross-linking efficiency of CMCS films, either through the incorporation of complementary compounds or by employing alternative cross-linking strategies.

## Figures and Tables

**Table 1:** Power-law model parameters of CS, CMCS, and their modified solutions.

	CS	CS/CTA	CS/PEGDE	CS/G1
***n***	1.06	0.86	1.13	1.15
***k*** (Pa·s)^n^	0.29	0.37	0.23	0.48
R^2^	1.00	0.996	1.00	1.00
	**CMCS**	**CMCS/CTA**	**CMCS/PEGDE**	**CMCS/G1**
***n***	0.82	0.86	0.69	0.62
***k*** (Pa·s)^n^	0.26	0.046	0.38	0.45
R^2^	0.998	0.996	0.985	0.982

***n***—the flow behavior index (dimensionless); ***k***—the consistency index.

**Table 2:** Roughness parameters of thin films with different compositions before and after NaOH treatment.

	R_q_ (nm)			
	CS	CS/CTA	CS/PEGDE	CS/G1
before	3.6 ± 0.7	1.7 ± 0.1	16.6 ± 1.3	1.5 ± 0.2
after	2.3 ± 0.6	12.0 ± 1.5	2.8 ± 0.7	0.8 ± 0.1
	**R_q_ (nm)**			
	**CMCS**	**CMCS/CTA**	**CMCS/PEGDE**	**CMCS/G1**
	0.3 ± 0.02	2.4 ± 0.5	24.1 ± 8.7	3.1 ± 0.9

**Table 3:** Sample codes and compositions.

Sample	CS (g)	CTA (g)	PEGDE (mmol)	G (mmol)
CS	0.7	−	−	−
CS/CTA	0.7	0.140	−	−
CS/PEGDE	0.7	−	0.300	−
CS/G2 CS/G1	0.7 0.7	− −	− −	0.375 0.120
	**CMCS (g)**	**CTA (g)**	**PEGDE (mmol)**	**G (mmol)**
CMCS	1.75	−	−	−
CMCS/CTA	1.75	0.263	−	−
CMCS/PEGDE	1.75	−	0.300	−
CMCS/G2 CMCS/G1	1.75 1.75	− −	− −	0.375 0.120

## Data Availability

All data are shown in the paper.

## Abbreviations

The following abbreviations are used in this manuscript:

CS	Chitosan
CMCS	Carboxymethyl chitosan
CTA	Citric acid
EB	Elongation at break
G	Glutaraldehyde
IR	Infrared spectroscopy
AFM	Atomic force microscope
PBS	Phosphate-buffered saline
PEGDE	Polyethylene glycol diglycidyl ether
R_q_	Root mean square
SEM	Scanning electron microscope
TGA	Thermogravimetric analysis
TS	Tensile strength
YM	Young’s modulus
